# An economic model of long-term use of celecoxib in patients with osteoarthritis

**DOI:** 10.1186/1471-230X-7-25

**Published:** 2007-07-04

**Authors:** Michael Loyd, Dale Rublee, Philip Jacobs

**Affiliations:** 1 Michael Loyd & Associates Ltd, Winnipeg, Manitoba, Canada; 2 Global Outcomes Research, Pfizer Inc, New York, USA; 3 University of Alberta, Edmonton, Alberta, Canada

## Abstract

**Background:**

Previous evaluations of the cost-effectiveness of the cyclooxygenase-2 selective inhibitor celecoxib (Celebrex, Pfizer Inc, USA) have produced conflicting results. The recent controversy over the cardiovascular (CV) risks of rofecoxib and other coxibs has renewed interest in the economic profile of celecoxib, the only coxib now available in the United States. The objective of our study was to evaluate the long-term cost-effectiveness of celecoxib compared with nonselective nonsteroidal anti-inflammatory drugs (nsNSAIDs) in a population of 60-year-old osteoarthritis (OA) patients with average risks of upper gastrointestinal (UGI) complications who require chronic daily NSAID therapy.

**Methods:**

We used decision analysis based on data from the literature to evaluate cost-effectiveness from a modified societal perspective over patients' lifetimes, with outcomes expressed as incremental costs per quality-adjusted life-year (QALY) gained. Sensitivity tests were performed to evaluate the impacts of advancing age, CV thromboembolic event risk, different analytic horizons and alternate treatment strategies after UGI adverse events.

**Results:**

Our main findings were: 1) the base model incremental cost-effectiveness ratio (ICER) for celecoxib versus nsNSAIDs was $31,097 per QALY; 2) the ICER per QALY was $19,309 for a model in which UGI ulcer and ulcer complication event risks increased with advancing age; 3) the ICER per QALY was $17,120 in sensitivity analyses combining serious CV thromboembolic event (myocardial infarction, stroke, CV death) risks with base model assumptions.

**Conclusion:**

Our model suggests that chronic celecoxib is cost-effective versus nsNSAIDs in a population of 60-year-old OA patients with average risks of UGI events.

## Background

Nonselective nonsteroidal anti-inflammatory drugs (nsNSAIDs) are widely used to treat acute and chronic pain, including the symptoms of osteoarthritis (OA). The most clinically important adverse events associated with nsNSAID use, ulcer perforations, obstructions, and bleeds (collectively POBs), can be life-threatening and constitute a major public health problem with a large cost to society [1-3]. Uncomplicated peptic ulcers and dyspepsia are not as important clinically as POBs, but, because they are the most prevalent nsNSAID adverse events, their total impact in healthcare costs, activity restrictions, production losses, pain, and discomfort is considerable [4-10].

The cyclooxygenase-2 selective inhibitor celecoxib reduces the risks of nsNSAID-induced gastropathy without compromising clinical efficacy. However, evaluations of the cost-effectiveness of celecoxib have produced conflicting results owing mainly to differences in model structures, populations at risk, probabilities, cost-effectiveness indicators, and relative drug prices across countries.

The objective of our study was to evaluate the cost-effectiveness of the long-term use of celecoxib compared with nsNSAIDs in a population of 60-year-old OA patients with average risks of upper gastrointestinal (UGI) complications who require chronic daily NSAID therapy. We assume that acetaminophen is contraindicated or otherwise inferior to NSAID therapy in these patients. Ours is the only coxib evaluation that uses UGI ulcer probabilities from a population comprised only of OA patients, and accounts for reductions in the prices of celecoxib and over-the-counter (OTC) omeprazole after loss of patent protection. Although there have been other lifetime coxib models, only ours extrapolates probabilities and models health states based on long-term evidence from the literature and evaluates celecoxib independently [11-13]. In sensitivity testing, the distinctive features of our study are its evaluations of a comprehensive indicator of serious cardiovascular (CV) thromboembolic risk, alternate treatment regimens after UGI adverse events, and differences in the lengths of the analytic horizon.

## Methods

Cost-effectiveness analysis is used to compare celecoxib (Celebrex, Pfizer Inc, USA) at 200 mg/d, the recommended OA dose, with a combination of diclofenac at 100 mg/d and naproxen at 1000 mg/d. The target population is 60-year-old patients with moderate to severe OA requiring chronic daily NSAIDs. The outcomes of the model are incremental costs per quality-adjusted life-year (QALY) gained from reductions in symptomatic peptic ulcers, perforations, obstructions, bleeding ulcers (collectively PUBs), and nonulcer dyspepsia. CV thromboembolic events and efficacy in relieving OA symptoms are excluded in the base model on the assumption that there are no differences in these outcomes between celecoxib and nsNSAIDs [14-17].

### Model

**Figure 1:**
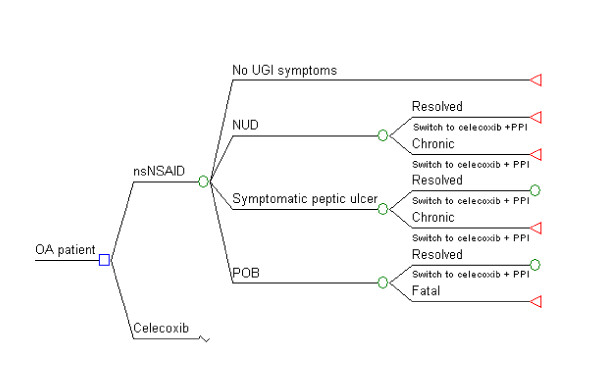
**Schematic representation of the study model**. nsNSAID, nonselective nonsteroidal anti-inflammatory drug; NUD, nonulcer dyspepsia-like composite; OA, osteoarthritis; PPI, proton pump inhibitor; POB, perforation, obstruction, bleeding ulcer; UGI, upper gastrointestinal.

The structure of the model used in this study is presented in Figure 1. The problem is structured in Microsoft Excel 2002 as a decision tree with 21 one-year periods. Our literature-based UGI adverse events were comprised of discrete and short-duration NUD, POB and peptic ulcer events in some patients and long-term chronic NUD and PUD health states in others. The model allowed for multiple POB and PUB events to recur in time for patients whose short-term discrete events were initially resolved. Patients on nsNSAIDs switch in perpetuity to celecoxib and patients in both cohorts add chronic daily omeprazole after experiencing nonulcer dyspepsia-like symptoms or a PUB event [12]. This assumption recognizes the heightened UGI risks after PUB events, which compound older patients' already high cumulative risks with long-term nsNSAID therapy, the risk that nonulcer dyspepsia might progress to a peptic ulcer, and the potential benefits from symptom relief [1,18,19]. In sensitivity analysis, we include 4 alternate post-event treatment approaches also used in clinical practice.

A lifetime analytic horizon is used because many OA sufferers are long-term NSAID users, with high cumulative risks of gastropathy, and some adverse events in the model affect health states for extended periods [20]. Our societal perspective, modified to exclude indirect costs, does not differentiate between patient and insurer costs and is compatible with our use of a lifetime horizon.

We assume that all members of the celecoxib and nsNSAID treatment groups are aged 60 years at the outset and all die in the base model at age 81 years, except those with an adverse gastrointestinal (GI) event resulting in premature death before the end of the 21-year lifetime horizon [12].

We conducted literature searches to obtain representative clinical adverse event rates and information on the long-term courses of health states after adverse events. We sought UGI adverse event rates for patients using the doses of celecoxib and nsNSAIDs recommended for OA patients. Celecoxib is more commonly used for the treatment of the symptoms of OA rather than rheumatoid arthritis. Our search strategies are outlined in more detail in Appendix I.

### Probabilities

Our probabilities for POBs and peptic ulcers are based on event rates in the Successive Celecoxib Efficacy and Safety Study-1 (SUCCESS), the only celecoxib clinical trial that was conducted in a population of OA patients and that used POBs and PUBs as end points [16]. This 12-week trial with a total of 13,274 patients allowed the use of concomitant aspirin (7.1% baseline prevalence) but excluded high-risk patients with active or recent GI disease, histories of multiple peptic ulcers, various other comorbidities, and those requiring corticosteroids or chronic gastroprotective agents. Patients with a previous history of ulcers comprised 4.1% of the SUCCESS population, about half the proportions in 2 other major coxib clinical trials, the Celecoxib Long-Term Arthritis Safety Study (CLASS) [21] and the Vioxx Gastrointestinal Outcomes Research study (VIGOR) [22].

The model's POB probabilities per 100 patient-years are 0.8 for nsNSAIDs and 0.1 for celecoxib, representing a crude relative risk reduction (RRR) for celecoxib of 0.875 [16]. This RRR is consistent with those from a pooled analysis of 14 arthritis clinical trials and a 42-week retrospective cohort study of hospitalized POBs [23,24]. Our symptomatic ulcer probabilities per 100 patient-years adapted from SUCCESS are 1.2 for nsNSAIDs and 0.9 for celecoxib (RRR = 0.25).

The base model extrapolates these probabilities over 21 years to calculate POB cumulative incidence rates of 15.5% for the naproxen-diclofenac combination and 2.1% for celecoxib, and respective peptic ulcer rates of 22.4% and 17.3% (Table 1) [24-29]. Our assumption that risks are constant over time, which is intended to produce a conservative base-case incremental cost-effectiveness ratio (ICER) for celecoxib, is relaxed in sensitivity testing to allow, more realistically, for increased risk with advancing age. Patients who experience a PUB event in the model are assumed to be 2.7 times more likely than others to suffer a future peptic ulcer or POB [18,25,28,30,31].

**Table 1:** Clinical probabilities

**Clinical probabilities**	**Base case (range)**	**Reference**
**Dyspepsia composite**		
nsNSAIDs	12.0% (9.9%–14.0%)	[32]
Celecoxib	7.8% (6.0%–9.5%)	[32]
**POB 21-year cumulative incidence**		
nsNSAIDs	15.5% (4.1%–24.8%)	[16,24-29]
Celecoxib	2.1% (0.0%–6.1%)	[16,24-29]
**Symptomatic peptic ulcer 21-year cumulative incidence**		
nsNSAIDs	22.4% (10.0%–33.7%)	[16,24-29]
Celecoxib	17.3% (10.0%–24.0%)	[16,24-29]
**PUB 21-year cumulative incidence**		
nsNSAIDs	34.6% (19.0%–47.3%)	[16,24-29]
Celecoxib	19.0% (10.0%–27.2%)	[16,24-29]
**Age-related increase in PUB risk per year**	4.3% (2.5%–6.1%)	[18,25,27,28,71,72]
**PUB risk multiplier for prior PUB event**	2.7 (1.5–4.7)	[18,25,28,30,31]
**Hospitalization rate for POBs**	90% (80%–100%)	[145-150]
**Mortality rate as percent of POBs**	8.0% (5.0%–14.0%)	[151-159]
**POB with prior dyspepsia**	35% (20%–50%)	[42,44,45]
**Ratio active ulcers and symptoms to lifetime-prevalent peptic ulcers**	50% (0%–65%)	See text
**Ratio chronic to lifetime-prevalent nonulcer dyspepsia**	55% (0%–75%)	[12,33,52,55,56]

nsNSAID, non-selective non-steroidal anti-inflammatory drugs; PUB, symptomatic peptic ulcers, perforations, obstructions, bleeding ulcers; POB, perforation, obstruction, bleeding ulcer; PPI, proton pump inhibitor.

The nonulcer GI adverse events of NSAIDs, consisting of dyspepsia (upper abdominal pain or discomfort), nausea, diarrhea, heartburn, constipation, and flatulence, are relevant to economic evaluations because they diminish patients' quality of life and result in increased treatment costs. However, we model only for dyspepsia-like symptoms (including nausea), mainly due to data limitations.

Our clinical probabilities for dyspepsia-like symptoms of 12.0% (95% confidence interval [CI], 9.9%–14.0%) for an nsNSAID and 7.8% (95% CI, 6.0%–9.5%) for celecoxib 200 mg/d are based on moderate to severe dyspepsia, abdominal pain, or nausea event rates in a pooled analysis of trials in arthritis patients (Table 1) [32]. We adopt these probabilities for our lifetime horizon based on findings that cumulative incidence curves for dyspepsia begin to plateau in trials, may be essentially flat beyond a year, and are unaffected by aging [32-34]. A second pooled analysis comparing OA patients taking celecoxib 200 mg/d or an nsNSAID found near-identical relative risks (RR) for a similar composite dyspepsia-like end point [35].

### QALYs, utilities, and durations of health states

We assume a base utility value of 0.67 for OA patients without UGI problems and apply utility adjustments for adverse events as deductions from the base (Table 2) [36,37]. The base utility value of 0.67 reflects the fact that the average health of older OA patients, who have a high prevalence of comorbid conditions, is considerably below perfect health, which carries a utility value of 1.0. The annual adjustments for adverse events are the product of the loss of utility from the adverse event and the length of the associated health state expressed as a fraction of a year.

**Table 2:** Utilities and QALY losses for simple health states

**Simple health states**	**QALY losses**	**Reference**
**Dyspepsia (-0.13)**		
Resolved events (35 days)	-0.01247 (-0.005753 to -0.01496)	[38]
Chronic per year	-0.13 (-0.06 to -0.156)	[38]
**Symptomatic peptic ulcers (-0.13)**		
Resolved events (35 days)	-0.01247 (-0.005753 to -0.01496)	[38]
Chronic per year	-0.13 (-0.06 to -0.156)	[38]
**Hospitalization stays**		
POB (ALOS = 5.56 days)	-0.007926 (-0.00634 to -0.009511)	[12,40,41]
Peptic ulcers (ALOS = 4.10 days)	-0.005868 (-0.004694 to -0.007042)	[12,40,41]
**Outpatient POB ER visit**	-0.001397 (-0.001118 to -0.001676)	[41]
**Endoscopy (-0.4325)**	-0.00119 (-000952 to -0.001428)	[41]
**Premature POB death per year**	-0.67 (-0.536 to -0.804)	[36,37]

QALY, quality-adjusted life year; POB, perforation, obstruction, bleeding ulcer; ALOS, average length of stay; ER, emergency room. QALY benefits discounted 3% annually.

Our disutilities of 0.13 for dyspepsia and peptic ulcers are based on individuals with moderate to severe dyspepsia aged 59 years or more [38]. We corroborated this peptic ulcer disutility by comparing differences in average Health Utility Index values for older OA patients with and without ulcers in a large Canadian database [39]. Disutilities for hospitalized cases were adapted from the literature, with the lengths of inpatient POB and peptic ulcer health states set at averages for such cases in a Maryland hospital database [40,41]. We factored in additional disutilities for 35% of POB patients with assumed prior symptoms of dyspepsia [42-45].

NSAID-associated nonulcer dyspepsia consists of single events, series of periodic events, or essentially continuous symptoms. Our modeling assumption is that 55% of dyspepsia patients experience chronic daily symptoms, whereas the remaining patients experience a single 35-day episode of symptoms [12,46-54]. The long-term proportion assumed to be chronic is consistent with the findings of 2 systematic reviews and other long-term studies [33,52,55,56].

The long-term course of peptic ulcer disease is complex, with high rates of peptic ulcer and ulcer-symptom recurrence and chronicity [4,12,46,54,57-59]. Our simplified modeling assumption is that the combined point prevalence of patients with active symptomatic ulcers and postulcer symptoms is 50% of lifetime prevalent ulcers developed over the analytic horizon of the model [60]. This means that the cumulative incidence rate in our model would average 6.1% over the analytic horizon in patients in the nsNSAID treatment arm compared with 4.7% in those in the celecoxib treatment arm.

To test this assumption, we analyzed the prevalence of ulcers among OA patients over the age of 59 years as determined by a Canadian population-based survey [39]. Seven percent of such patients reported having a current physician-diagnosed ulcer of at least 6 months' duration. This means that the steady state prevalence in a population of OA patients in the community, who are not necessarily NSAID users, exceeds the average prevalence for NSAID users in our base model.

### Costs and other data

We assume that generic celecoxib will be available in 2013, the year of patent expiry, and that the price will be 55% lower than that of Celebrex, with a range of 30% to 80% for sensitivity testing (Table 3) [61-64]. We employ only the estimated generic price in our model after mid 2013. This is consistent with our approach of using prices of generic nsNSAIDs and OTC omeprazole in the model.

**Table 3:** Cost* and other data summarized

	**Base case (range)**	**Reference**
**Celecoxib 200 mg/d**	$2.64 ($2.11–$3.17)	
**Naproxen 1000 mg/d**	$0.45 ($0.36–$0.54)	
**Diclofenac 100 mg/d**	$0.64 ($0.51–$0.77)	
**Omeprazole (over-the-counter) 20 mg/d**	$0.60 ($0.48–$0.72)	
**Price reduction generic celecoxib**	55% (30%–80%)	[61,63]
**POB inpatient-hospital plus physician (ALOS = 5.56 days)**	$12,796 ($10,234–$15,355)	[12,40]
**POB outpatient-hospital plus physician**	$1,813 ($1,450–$2,176)	[12,160,161]
**Peptic ulcer inpatient-hospital plus physician (ALOS = 4.10 days)**	$7,353 ($5,882–$8,824)	[12,40]
**Peptic ulcer outpatient discrete event including endoscopy**	$1,554 ($1,243–$1,865)	[12,160,161]
**Dyspepsia outpatient initial event including endoscopy**	$1,208 ($966–$1,450)	[12,160,161]
**Ongoing annual medical and laboratory costs chronic peptic ulcer or dyspepsia**	$130 ($104–$156)	[160], Estimates RBRVS
**POB proportion of surgery DRGs**	14.2%	[40]
**POB admissions through emergency**	89.7%	[40]
**Proportion of peptic ulcer patients admitted to hospital**	4.37%	[40]
**Discount rate for costs and QALYs**	3.0%	
**Price inflation rate**	2.0%	

POB, perforation, obstruction, bleeding ulcer; ALOS, average length of stay; RBRVS, resource-based relative value scale; DRGs, diagnosis-related groups. *Drug prices as of February 2006.

Average inpatient hospital costs, lengths of stay, and various hospital statistics were derived from a Maryland inpatient database [40]. We employed principal diagnosis codes and diagnosis-related groups to identify relevant cases and restricted our analyses to patients aged 60 years or more. In the Maryland system, each hospital's charges are based on standardized overhead allocation methods, regulated to reflect the costs (including capital) of services provided, and charge rates apply to all payers without discrimination or discounting. The average cost per adjusted admission in Maryland approximates the national average.

## Results

Incremental treatment costs and benefits of $4,055 and 0.1304 QALYs resulted in a base-case celecoxib versus nsNSAID ICER of $31,097 per QALY, which falls within the range normally considered to be cost-effective (Table 4). The 21-year treatment costs for average patients in the celecoxib and nsNSAID initial treatment groups were $14,151 and $10,096, respectively, whereas the corresponding QALYs were 10.2982 and 10.1678.

**Table 4:** Base model ICER

	**QALYs**	**Costs**	**ICER**
**nsNSAID**	10.1678	$10,096	
**Celecoxib**	10.2982	$14,151	
**Net incremental**	0.1304	$4,055	$31,097

ICER, incremental cost-effective ratio; QALY, quality-adjusted life year; nsNSAID, nonselective nonsteroidal anti-inflammatory drugs.

### Sensitivity testing

#### Uncertainty

One-way sensitivity analysis results for clinical and economic variables showed that the ICER rose above $60,000 per QALY (our standard for cost effectiveness) for only 2 of the variables when ranged over the values in Tables 1, 2, or 3, the nsNSAID peptic ulcer and POB probabilities [65]. Other relatively influential variables in 1-way analyses were (in descending order of influence): the celecoxib price, and the event probabilities for a celecoxib peptic ulcer, nsNSAID dyspepsia, and a celecoxib POB.

Univariate analysis of the sensitivity of the ICER to proportional changes in economic and clinical variables identified the following important variables listed in declining order of their impact on the ICER, along with their threshold values and the proportional changes in each required to attain threshold: celecoxib cost per pill, $3.72 (+41%) and 21-year probabilities of nsNSAID dyspepsia, 0.0743 (-38%); nsNSAID peptic ulcer, 0.126 (-44%); nsNSAID POB, 0.061 (-61%); and celecoxib dyspepsia, 0.1295 (+66%). Hence, the results are robust for all but very large proportional changes.

In addition to the above sensitivity analysis for uncertainty, we assessed variants of the base model in which: 1) risks of PUBs increase with age; 2) the length of the analytic horizon is varied; 3) treatment regimens are altered after adverse events; and 4) different serious CV thromboembolic risks are assumed.

#### Risks with aging

Advanced age is a well-documented risk factor for PUBs [1,3,31,66-70]. In this sensitivity analysis, we assumed that PUB risks increase by 4.3% per year after age 60 years [18,25,27,28,71,72]. The assumption implies that PUB risks at age 81 years are 2.3 times those at baseline for patients aged 60 years.

The ICER declined to $19,309 when age-related increases in PUB risks were factored into the base-model assumptions (Table 5). The cumulative incidence rates were 28.3% for PUBs in the celecoxib initial treatment group and 48.9% in nsNSAID group, with corresponding POB cumulative incidence rates of 3.3% and 23.4%, respectively. When dyspepsia was included, about 60.9% of nsNSAID patients had at least 1 UGI adverse event over the analytic horizon compared with 36.1% of celecoxib patients.

**Table 5:** Summary of base model and special sensitivity analysis results

**Model features**	**No age risks**	**Sensitivity analyses with age risks**
**Lifetime models**		
Base model	$31,097	$19,309
Alternative treatments after UGI events models	$38,807–$46,192	$26,201–$31,777
CV thromboembolic risks 1^[15]^	$17,120	$7,923
**Other analytic horizons**		
1 year	$124,100	$124,100
5 years	$108,549	$104,104
8 years	$93,420	$85,534
11 years	$67,812	$58,459
12 years	$61,191	$51,521

UGI, upper gastrointestinal; CV, cardiovascular.

#### Analytic horizon

The ICER declined 74.9% from $124,100 for a 1-year model to $31,097 for the 21-year model without age risks (Table 5). In the absence of the patent expiration effect, the ICER would have decreased 53.8% from $124,100 to $57,363 in a 21-year model. The ICER declined below $60,000 per QALY as of year 13 of the analytic horizon in the base case, and year 11 in the sensitivity analysis in which the risk of a PUB event increased with advancing age.

#### Alternative treatment approaches after a UGI adverse event

The base model switched patients initially taking celecoxib or an nsNSAID to regimens of celecoxib plus a proton pump inhibitor (PPI) after dyspepsia or a PUB, an approach that is clinically reasonable given the elevated risks of PUBs and perhaps death after such events. In sensitivity analysis, we also assessed 4 alternate secondary strategies to determine whether the ICER for celecoxib versus an nsNSAID would remain below $60,000 per QALY for other possible postevent treatment regimens.

The 4 alternate treatment strategies were: 1) all patients continue on their original treatment regimen, after undergoing temporary courses of PPI healing therapy for PUBs and *Helicobacter pylori *eradication therapy, as necessary; 2) all patients continue on celecoxib or their nsNSAID and commence PPI cotherapy for the rest of their lives after PUBs; 3) all nsNSAID patients switch to celecoxib plus PPI and celecoxib patients add a PPI for the rest of their lives post-PUB, but nonulcer dyspepsia patients continue on their original treatment regime; and 4) all nsNSAID patients add a PPI after PUBs with all other treatments unchanged after events.

Celecoxib is cost-effective at the sub-$60,000 per QALY level for models incorporating each of the 4 post event treatment regimens (Table 5) [37,50,73-77]. Clinical probabilities were not sufficiently robust to support a full comparison of ICERs between the alternate regimes.

#### Depletion of susceptibles

We also tested an alternate assumption that our SUCCESS POB probabilities were too high for constant-risk extrapolation in our model owing to depletion of susceptibles, the theory that risks are higher in the first few months of NSAID use. The ICER in the base case rose to $40,841 per QALY in a base case model with a 30% reduction in POB risks. The ICER further rose to $45,955 per QALY when we extended the 30% reduction to peptic ulcer probabilities and POBs.

#### Risks of serious CV thromboembolic events

Our serious CV thromboembolic event (myocardial infarction [MI]), stroke, CV death) costs, and disutilities take into account the impact of the initial event and of subsequent effects, as described in more detail in Appendix II.

Our probabilities of such events are based on a meta-analysis of serious CV thromboembolic risk that found event rates of 1.01 per 100 patient-years in celecoxib patients and 1.23 per 100 patient-years in nsNSAID users, with a RR of 0.86 (95% CI, 0.59–1.26) [15]. The ICER in our model declines to $17,120 per QALY when these risks are included, and our results were robust when the RR was varied over all but the extreme upper end of the 95% CI.

## Discussion

We conducted a cost-effectiveness analysis of long-term celecoxib use compared with nsNSAID use in OA patients. Some of our key assumptions, not adequately addressed in previous models, include a more realistic analytic horizon for UGI adverse events in OA patients with extrapolation and health states based on long-term evidence from the literature, prescription drug prices influenced by termination of patent protection, and ulcer probabilities derived from an OA population.

Our base-case celecoxib ICER of $31,097 per QALY suggests cost-effectiveness for 60-year-old OA patients with average baseline UGI risks, 7.1% of whom were taking aspirin for cardioprophylaxis. The ICER declines to $19,309 when account is taken of the increase in PUB risks with advancing age. The ICER, including risks of serious CV thromboembolic events, is $17,120 for a model without age-related risks, and ICERs ranged from $38,807 to $46,192 per QALY in sensitivity analysis with alternate post adverse event treatment strategies.

Our model suggests that the use of short analytic horizons to evaluate long-term celecoxib therapy in OA patients results in biased findings [20,78,79]. The diverging cumulative incidence of PUBs in patients in our model's 2 initial treatment groups results in progressively larger differences between comparators' incidences of subsequent PUB events, their ongoing disutilities from chronic peptic ulcers, and their continuing expenses from higher-cost post-PUB treatment regimens. Additionally, truncated horizons neither capture the full QALY impact of the higher mortality rates in nsNSAID patients nor take into account future celecoxib patent expiration and the related price decreases. Finally, if the risk of PUBs increases with advancing age, as the evidence suggests, the impact of this factor would be muted by short analytic horizons.

Previous coxib evaluations do not appear to have accounted for the patent expiration of celecoxib in 2013 or that of omeprazole in 2002, although PPIs are important drivers of costs in some models and the current OTC price represents a reduction of more than 75% in the United States.

Our main results are based on regimens of daily nsNSAIDs, whereas intermittent therapy provides adequate relief of OA symptoms for some users of chronic nsNSAIDs in the community. Additionally, some candidates for continuous nsNSAID therapy may interrupt treatment periodically as a strategy to lower MI and stroke risks, even though they suffer disutilities from symptom relief foregone during washout periods. Published coxib evaluations have assumed continuous nsNSAID use because evidence-based probabilities for intermittent treatment regimens are unavailable and myriad patterns of intermittent use are possible [80].

We assessed frequent but intermittent nsNSAID use, which we defined as a minimum of 104 days of therapy per year or approximately 2 days per week for 52 weeks. Our celecoxib versus nsNSAID ICERs were $43,000 per QALY or lower depending on the assumed behavior of UGI risks during intermittent use, number of therapy days, and other factors. Hence, our model's results are not contingent on a restrictive assumption of continuous use.

Six evaluations from Europe, Asia, and Canada suggest a favorable economic profile for celecoxib [13,81-85]. However, 5 of these studies with a similar evaluation framework incorporate nsNSAID ulcer probabilities considerably higher than those in the major trials, and international differences in drug prices and health system costs also limit the scope for generalizing these findings to other countries [16,21,22]. Two American studies suggest that celecoxib may be cost-effective compared with an nsNSAID [86,87].

Conversely, an influential American study evaluating a celecoxib-rofecoxib hybrid and a Canadian study comparing celecoxib with ibuprofen and diclofenac produced base-case ICERs far outside ranges considered to be cost-effective, although both found that coxibs might be cost-effective in high-risk populations [12,37]. These 2 studies of OA and rheumatoid arthritis patients not taking low-dose aspirin relied on controversial longer-term follow-up findings from the CLASS trial for critical assumptions [88-95]. These assumptions from CLASS, the earliest celecoxib trial with both POBs and PUBs as end points, conflict with current evidence, including findings from subsequent coxib trials.

Differences in assumptions and model parameters created most of the inconsistencies between the results of the American study by Spiegel and coauthors and our findings. Our model used the SUCCESS trial results as the basis for the key celecoxib and nsNSAID ulcer probabilities, whereas Spiegel used those for a coxib hybrid with predominant weightings from rofecoxib trials and CLASS, with its supratherapeutic doses of celecoxib. More important, Spiegel's results were driven by an assumption that the annual new patient incidence of PUBs would decrease by 35% per year over its entire 21-year analytic horizon. This pivotal assumption was based on a special-case comparison of the POB incidence rates of nsNSAID patients in the first 6 months and the remaining observational period of the CLASS trial [12]. Under the assumed decay function, the cumulative incidence rate of PUBs in nsNSAID patients was 2.6% in the first year and totaled only 7.2% in the 21-year horizon of the Spiegel model. In contrast, the 21-year incidence rate of 34.6% in our base model was almost triple the upper end of the range in Spiegel's sensitivity test. The differences in the ulcer probabilities are the most important difference between the Spiegel model and the model reported here. Other limitations of the Spiegel model are that it did not take into account any of the following: increases in UGI risks from the aging of its cohorts over the 21-year analytic horizon, increased UGI risks after PUB events, and the price reductions from the expiries of patents on celecoxib and omeprazole.

The data in our review of coxib and nsNSAID trials ranging in duration from 1 to 3 years support the generalization that PUB and POB risks are constant in long clinical trials [22,30,31,96,97]. Hence, we believe that the weight of previous and newer trial evidence supports the conclusion of the CLASS authors and others that the POB and PUB event rates observed in nsNSAID patients in the longer follow-up portion of CLASS are biased and that the decline in POB rates in this trial was aberrant [66,88,93,94,98]. The long-term decay function in the Spiegel model also conflicts with evidence from observational studies ranging in length from 2 to 15 years [25-29,99-102].

The use of ulcer event rates from the longer-term CLASS trial is 1 reason for the inconsistency of Maetzel and colleagues' Canadian model with our results [37,80]. The RRRs in PUB rates range from 47% to 50% for coxibs versus nsNSAIDs in typical trials [16,22,96,97]. However, for celecoxib compared with diclofenac and ibuprofen, Maetzel's PUB RRs from the CLASS trial were 0.975 and 0.362, respectively (versus our 0.50 compared with nsNSAIDs) and his POB RRs were 0.917 and 0.386, respectively (versus our 0.125 compared to nsNSAIDs) [37,80]. Additionally, Maetzel assumed relative dyspepsia risks of 1.00 (versus our 0.65) and relative MI risks of 1.39 to 1.44 (versus our equality of serious CV thromboembolic event risks). Other important differences between the Maetzel model and ours are its 5-year horizon and absence of patent expiration effects, lower probabilities of death from initial compared with repeat POBs, and withdrawal of 95% of POB patients from further NSAID treatment [37,80].

Probabilities obtained from the SUCCESS trial and our modeling decisions tend to be conservative. Relevant decisions include the assumed independence of PUB risks and age in the base model and the exclusion of indirect costs. The doses used in the SUCCESS trial tended to favor nsNSAIDs: one half the celecoxib patients received double the dose recommended for OA patients, whereas the 100-mg daily diclofenac dose was less than the maximum dose approved for OA patients in participating countries [16,18,21,23,103]. Also, diclofenac, the main comparator in the SUCCESS trial, is one of the less toxic nsNSAIDs [104,105].

The results of our model cannot necessarily be generalized to all populations of OA patients. Furthermore, our results are contingent both on the validity of the short-term probabilities in the model and of our approach to extrapolating them over a 21-year period. Although extrapolation is commonly employed in, and generally recommended for, cost-effectiveness analyses of pharmaceuticals, it engenders uncertainties that increase with the length of the period [106].

According to 3 competing theories, CV thromboembolic risks may be associated with individual NSAIDs, coxibs as a class, or all nonaspirin NSAIDs as a class. Incremental cost-effectiveness analysis as employed in this article deals only with differences in risks between nsNSAIDs and celecoxib.

One limitation of the clinical trials in which celecoxib versus nsNSAID CV thromboembolic events have been studied to date, including those in the meta-analysis we used to account for possible differences in CV risks, is that none were originally designed for the primary purpose of assessing differences in these events between treatment groups and they tend to be underpowered for this purpose. Furthermore, the follow-up periods in most of the clinical trials in the meta-analysis that we used were shorter than one year in duration. However, longer observational studies of CV risk are consistent with a similarity of celecoxib and nsNSAID risks, even though they often employ MI end points, which generally favor nsNSAIDs in clinical trials [107-121]. Current evidence about relative CV thromboembolic risk is controversial and does not exclude the possibility that celecoxib at OA doses is associated either with elevated or decreased risks of CV thromboembolic events versus nsNSAIDs.

## Conclusion

Our model suggests that the long-term use of celecoxib is cost-effective versus nsNSAIDs in a population of 60-year-old OA patients with average risks of UGI events.

## Competing interests

This study was supported by Pfizer Inc, New York, USA. Dale Rublee is a full-time employee of Pfizer Inc. He holds stock options in Pfizer. Michael Loyd & Associates Ltd has consulting agreements with Pfizer Inc for this study and a related study. He owns Pfizer stock. Philip Jacobs received honoraria from Pfizer Inc and Michael Loyd & Associates Ltd.

## Authors' contributions

All authors contributed to the design, conduct and analysis of the study, modeling decisions, and the preparation of the manuscript. All authors read and approved the final manuscript.

## Appendix 1 – literature search strategy

To obtain clinical probabilities for symptomatic ulcers and POBs, we searched PUBMED for all clinical trials comparing celecoxib versus nsNSAIDs at recommended doses for the treatment of OA patients with symptomatic peptic ulcers or ulcer complications as end points. Key words used in the search were "clinical trial" or "RCT" (randomized clinical trial); "osteoarthritis"; "celecoxib," "ulcer," "hemorrhage," "haemorrhage," "bleed," "bleeding," "perforation," "POB," "PUB", or "PUD" (peptic ulcer disease). We reviewed the resultant abstracts, obtained the full articles as warranted, and reviewed article references. SUCCESS was the only clinical trial with the required end points that exclusively involved OA patients. The SUCCESS trial partially fulfilled our dosage objective, but one half of the celecoxib patients received double the 200-mg daily dose recommended for OA patients.

Articles were identified on 2 other clinical trials with some relevance, but both included OA and rheumatoid arthritis patients and also proved to be inferior matches in other respects [21,23]. We excluded the CLASS study from all PUB analyses owing to controversies associated with the study's ulcer-related results, and its use of 800 mg/d celecoxib doses, which are quadruple the dose recommended for OA patients. We compared POB RRs from SUCCESS with those from the second best match, an article on a pooled study of randomized clinical trials. However, this article did not report event rates for symptomatic peptic ulcers or PUBs.

We also searched for observational studies that most closely matched our criteria, given the dearth of relevant celecoxib clinical trial data to compare with SUCCESS probabilities. We wanted insights into adverse event rates in the community, and POB or PUB rates over longer periods. Two case control studies involving celecoxib were identified, one with average-risk patients and another with high-risk patients [24,122]. Only the former was pertinent to the current analysis.

For clinical probabilities of dyspepsia, we sought articles on clinical trials involving OA patients and comparing nsNSAIDs and celecoxib at doses normally used in OA patients, with relatively comprehensive definitions of dyspepsia, including related UGI symptoms, but excluding heartburn as a dominant symptom. Our search used key words "clinical trial" or "RCT," "dyspepsia," "abdominal pain," "UGI discomfort" or "NUD," "celecoxib," "NSAIDs," and "arthritis." We identified 3 articles, but the article based on CLASS was eliminated because supratherapeutic doses of celecoxib were used [32,34,35].

Various other searches were conducted including systematic searches on PubMed for all articles on the risks and courses of dyspepsia, peptic ulcers, POBs, strokes and MIs over extended periods. The objectives were to determine, based on the best available evidence, how to model the period prevalence rates, annual incidence rates, and durations of events over our analytic horizon, which extends far beyond the durations of clinical trials. We searched the literature systematically and manually for multivariate analyses of the risk factors for peptic ulcers and POBs, focusing especially on analyses with age as a continuous variable or with multiple categorical age interval variables. We also systematically reviewed other analyses powered to provide breakdowns of RRs by age.

The searches for UGI events were last conducted in January 2005.

## Appendix 2 – costs and disutilities of CV thromboembolic events

Health sector costs for first MIs or strokes included initial hospitalizations, ongoing maintenance costs, and forecasted excess costs of future care [40,123-130]. Among survivors, QALY decrements consisted of losses from initial hospital stays, ongoing postevent disutilities of 0.05 for MI survivors and 0.25 for stroke survivors, plus loss of life expectancy assuming that mortality rates by age for survivors were double the rates for the general population [37,39,131-141]. The impacts of first-in-model events on patients with a history of MI or stroke at baseline reflected patients' premodel reductions in life expectancy, utilities, and higher health sector costs [142-144]. The risks of CV thromboembolic events were assumed to increase 5% per year in sensitivity analyses that also assumed age-related increases in the risks of UGI adverse events.

## Pre-publication history

The pre-publication history for this paper can be accessed here:


